# The Development and Validation of the Smell‐Qx Questionnaire, Based on a Systematic Review of the Literature and the COMET Initiative on the Development of Core Outcome Sets for Clinical Trials in Olfactory Disorders

**DOI:** 10.1002/alr.23604

**Published:** 2025-05-09

**Authors:** Matt Lechner, Alexander Fjaeldstad, Umar Rehman, Jacklyn Liu, David Boniface, Jim Boardman, Duncan Boak, Aytug Altundag, Johannes Frasnelli, Simon Gane, Eric Holbrook, Julien Hsieh, Caroline Huart, Iordanis Konstantinidis, Basile N. Landis, Valerie J. Lund, Alberto Macchi, Eri Mori, Christian Mueller, Joaquim Mullol, Simona Negoias, Zara M. Patel, Jayant M. Pinto, Sophia Poletti, Vijay Ramakrishnan, Philippe Rombaux, Jan Vodicka, Antje Welge‐Luessen, Katherine L. Whitcroft, Carol Yan, Carl Philpott, Thomas Hummel

**Affiliations:** ^1^ Division of Surgery and Interventional Science UCL London UK; ^2^ UCL Cancer Institute London UK; ^3^ Department of Otorhinolaryngology Hospital Unit West Central Denmark Region Hospital Unit West Holstebro Denmark; ^4^ Department of Clinical Medicine Aarhus University ‐ Øre‐ Næse‐ og Halskirurgi RHG Aarhus N Denmark; ^5^ UCL London UK; ^6^ Fifth Sense Bicester UK; ^7^ Department of Otolaryngology Head and Neck Surgery Biruni University School of Medicine Istanbul Turkey; ^8^ Department of Anatomy Université du Québec à Trois‐Rivières Trois‐Rivières Quebec Canada; ^9^ Royal National Ear Nose Throat and Eastman Dental Hospitals London UK; ^10^ Massachusetts Eye and Ear Boston USA; ^11^ Department of Otolaryngology Head and Neck Surgery Harvard Medical School Boston USA; ^12^ Department of Otorhinolaryngology‐Head and Neck Surgery Geneva University Hospitals Geneva Switzerland; ^13^ Department of Otorhinolaryngology Head and Neck Surgery Cliniques Universitaires Saint‐Luc Brussels Belgium; ^14^ 2nd Academic ORL Department Aristotle University of Thessaloniki Thessaloniki Greece; ^15^ ENT Department ASST Sette Laghi University of Insubria Varese Italy; ^16^ Department of Otorhinolaryngology Jikei University School of Medicine Tokyo Japan; ^17^ Department of Otorhinolaryngology Head and Neck Surgery Medical University of Vienna Vienna Austria; ^18^ Department of Otorhinolaryngology Hospital Clínic Barcelona Barcelona Spain; ^19^ Department of Otorhinolaryngology‐Head and Neck Surgery Inselspital Bern University Hospital University of Bern Bern Switzerland; ^20^ Department of Otolaryngology—Head and Neck Surgery Stanford University School of Medicine Stanford California USA; ^21^ Department of Surgery The University of Chicago Chicago Illinois USA; ^22^ Department of Otorhinolaryngology Smell and Taste Clinic Technische Universität Dresden Dresden Germany; ^23^ Department of Otolaryngology Head and Neck Surgery Indiana University School of Medicine Indianapolis Indiana USA; ^24^ Department of Otorhinolaryngology Cliniques Universitaires Saint Luc Université Catholique de Louvain Brussels Belgium; ^25^ Department of Otorhinolaryngology and Head and Neck Surgery Regional Hospital Pardubice and Faculty of Health Studies University of Pardubice Pardubice Czech Republic; ^26^ Department of Otorhinolaryngology Head and Neck Surgery University Hospital Basel Basel Switzerland; ^27^ UCL Ear Institute University College London London UK; ^28^ Sheffield Children's Hospital Sheffield UK; ^29^ Department of Otolaryngology Head and Neck Surgery University of California San Diego School of Medicine La Jolla California USA; ^30^ University of East Anglia Medical School University of East Anglia Norwich UK; ^31^ Ear Nose and Throat (ENT) Department James Paget University Hospital James Paget University Hospitals NHS Foundation Trust Great Yarmouth UK

**Keywords:** gustatory dysfunction, olfactory dysfunction, patient‐reported outcome measures

## Abstract

**Background:**

Olfactory dysfunction affects up to 22% of the population. Accurate assessment is vital for diagnosis and tracking outcomes, often using patient‐reported outcome measures (PROMs).

**Aims:**

We aimed to develop and validate a novel questionnaire for assessing olfactory and gustatory dysfunction.

**Methods:**

A systematic review identified existing smell and taste questionnaires, followed by item generation and selection. After two Delphi cycles and consultation with a large panel of smell and taste experts, the Smell‐Qx questionnaire was developed. A validation study recruited patients from smell and taste clinics (cases) and general ENT clinics (controls) to complete the Smell‐Qx. Additionally, patients with smell and taste disorders underwent psychophysical testing using Sniffin’ Stick Threshold, Discrimination, and Identification (TDI) tests.

**Results:**

The Smell‐Qx is an 11‐domain instrument, with five core domains used for total score calculation and six history/quality‐of‐life domains for obtaining a comprehensive history. The validation study recruited 60 participants (32 patients with smell/taste disorders and 28 controls). Items showed acceptable to significant internal consistency (Cronbach's *α*: 0.64–0.97) and test–retest reliability (ICC: 0.65–0.99, *p* < 0.001). The Smell‐Qx was effective at distinguishing patients with smell and taste disorders from controls (*t* = 9.99, df = 58, *p* < 0.0001). Concurrent criterion validity was good with overall SATD‐related quality of life (*r* = 0.43, *p* = 0.015), as well as with the smell loss domain and overall smell TDI scores (*r* = −0.54, *p* = 0.011).

**Conclusion:**

The Smell‐Qx is a reliable and valid PROM for assessing olfactory and self‐reported gustatory disorders, capturing symptom severity and quality‐of‐life impact. It can integrate into a multi‐modal assessment approach alongside psychophysical testing.

## 1 Introduction

Smell and taste disorders (SATDs) are increasingly recognized, with olfactory dysfunction affecting around 22% of the population [1, 2, 3, 4, 5], and gustatory dysfunction affecting up to 17.3%, often alongside olfactory disorders [6]. The burden of these dysfunctions has increased since the COVID‐19 pandemic [7, 8]. Smell and taste are essential for food enjoyment, social interaction, safety, and mental well‐being [1, 2].

Olfactory and gustatory dysfunctions are categorized into quantitative and qualitative disorders [2]. Quantitative disorders impact the detection and identification of odors/tastants (e.g., hyposmia, anosmia, hypogeusia, and ageusia), while qualitative disorders affect odor quality (e.g., parosmia, phantosmia, olfactory intolerance, parageusia, and phantageusia) [2, 3]. It is frequent for both quantitative and qualitative disorders to coexist in affected individuals [2, 3].

Accurate assessment of olfactory and gustatory function is crucial, as subjective assessments poorly correlate with psychophysical testing, and to monitor patient outcomes over time [9]. Evaluation methods include subjective, patient‐reported measures, psychophysical tests, and objective techniques like electrophysiology and fMRI [2, 3].

Psychophysical tests like the Sniffin’ Sticks Smell Threshold, Discrimination, and Identification (TDI), the Smell Identification Test 40 (SIT40), and others are valuable for measuring olfactory function and identifying quantitative disorders [10, 11, 12]. However, they do not capture the impact of olfactory dysfunction on daily life or assess qualitative dysfunction [2, 3, 4]. This highlights the need for patient‐reported outcome measures, such as olfactory‐specific questionnaires, which help identify individuals with olfactory challenges [1, 2, 3, 4]. However, existing questionnaires have been criticized for their limited ability to assess the full range of qualitative disorders [3, 13].

Gustatory dysfunction is less common than olfactory dysfunction [3, 14]. Perception of complex flavors relies on retronasal olfaction, while true taste focuses on sweet, sour, salty, bitter, and umami [15]. When patients report a loss of taste, they may actually refer to a loss of flavor perception, typically linked to olfactory disorders, though this is often not considered in questionnaires focused on olfactory function [3].

### 1.1 Aims

The primary aim of this study was to develop and validate a novel, disease‐specific patient‐reported outcome measure for olfactory and gustatory disorders (SATDs) that addresses qualitative and quantitative olfactory and gustatory dysfunction, functional limitations, the impact on quality of life (QOL), and other emotional consequences.

## 2 Materials and Methods

### 2.1 Study Design

#### 2.1.1 Systematic Review

A systematic literature search was performed using PubMed in May 2023. Only original research studies focusing on the validation of olfactory or gustatory dysfunction questionnaires were included. The search terms are listed in Table S1. Two independent reviewers (J.L. and U.R.) screened titles and abstracts for eligibility, then reviewed full papers before inclusion.

#### 2.1.2 Item Generation and Preliminary Item Selection

A total of 384 items were extracted from surveys, categorized as diagnostic (e.g., identifying the symptom), assessing symptom extent (e.g., duration of smell loss), or evaluating symptom impact (e.g., effects on quality of life and other health‐related factors). Duplicates or similar items were removed. Item selection followed established olfactory terminology [2] and the Core Outcome Measures in Effectiveness Trials (COMET) initiative's core outcome was set for olfactory disorder clinical trials [16]. Five experts (M.L., A.F., T.H., V.L., and C.P.), with input from the Clinical Olfactory Working Group (COWoG) and members of Fifth Sense (UK charity for those with SATDs, Bicester, Oxfordshire, UK) [17], led this refinement with support from the research team.

#### 2.1.3 Delphi Cycle 1

During the first cycle of the Delphi process, 28 individuals including COWoG members and other experts reviewed the identified items and gave a score between 1 and 9 to each question in each section (1–3: unimportant; 4–6: moderately important; 7–9: essential). This generated the first iteration (items most useful for a targeted and time‐efficient assessment of a patient's sense of smell and taste). These consisted of two parts: (1) a history component and (2) the presence and extent of olfactory and gustatory dysfunction, including self‐reported ratings and impact on quality of life.

#### 2.1.4 Delphi Cycle 2

In May 2023, at the Smell and Taste Symposium in Norwich, UK, a panel of 31 experts, including rhinologists, ENT surgeons, physicians, and manuscript authors, reviewed the Smell‐Qx. Feedback from Patient Public Involvement (Fifth Sense) [16] led to further refinement, and the survey's first iteration was evaluated for content validity. Following further feedback, a second Delphi cycle was conducted, with COWoG members and experts scoring each question from 1 to 9 (1–3: unimportant; 4–6: moderately important; 7–9: essential). This process finalized the Smell‐Qx, comprising 11 domains—such as olfactory loss, parosmia, gustation, quality of life, and satisfaction with life—totaling 70 items (Supporting Information Appendix 2).

Each domain includes sub‐items beginning with a yes/no question; a severity scale follows if the response is affirmative. Follow‐up questions assess symptom progression, frequency and severity via Likert scales.

#### 2.1.5 Smell‐Qx Score Calculation

The total Smell‐Qx score was termed the APPOT Score and was calculated by rating each symptom or sensory domain from 0 to 5, resulting in a total score out of 25. The scoring for each symptom or sensory domain is as follows:

- Anosmia/hyposmia: 0–5.
- Parosmia: 0–5.
- Phantosmia: 0–5.
- Olfactory intolerance: 0–5.
- Taste: 0–5.

Total APPOT Score: /25

#### 2.1.6 Quality of Life Scores

Overall SATD‐related quality of life consisted of a single item, scored on a scale from 0 (no problem) to 5 (worst problem). The overall general QOL score incorporated various subdomains—social relationships, food, emotions, and hygiene—comprising 21 items, each rated individually on the same 0 to 5 scale, with a maximum combined score of 105.

**FIGURE 1:**
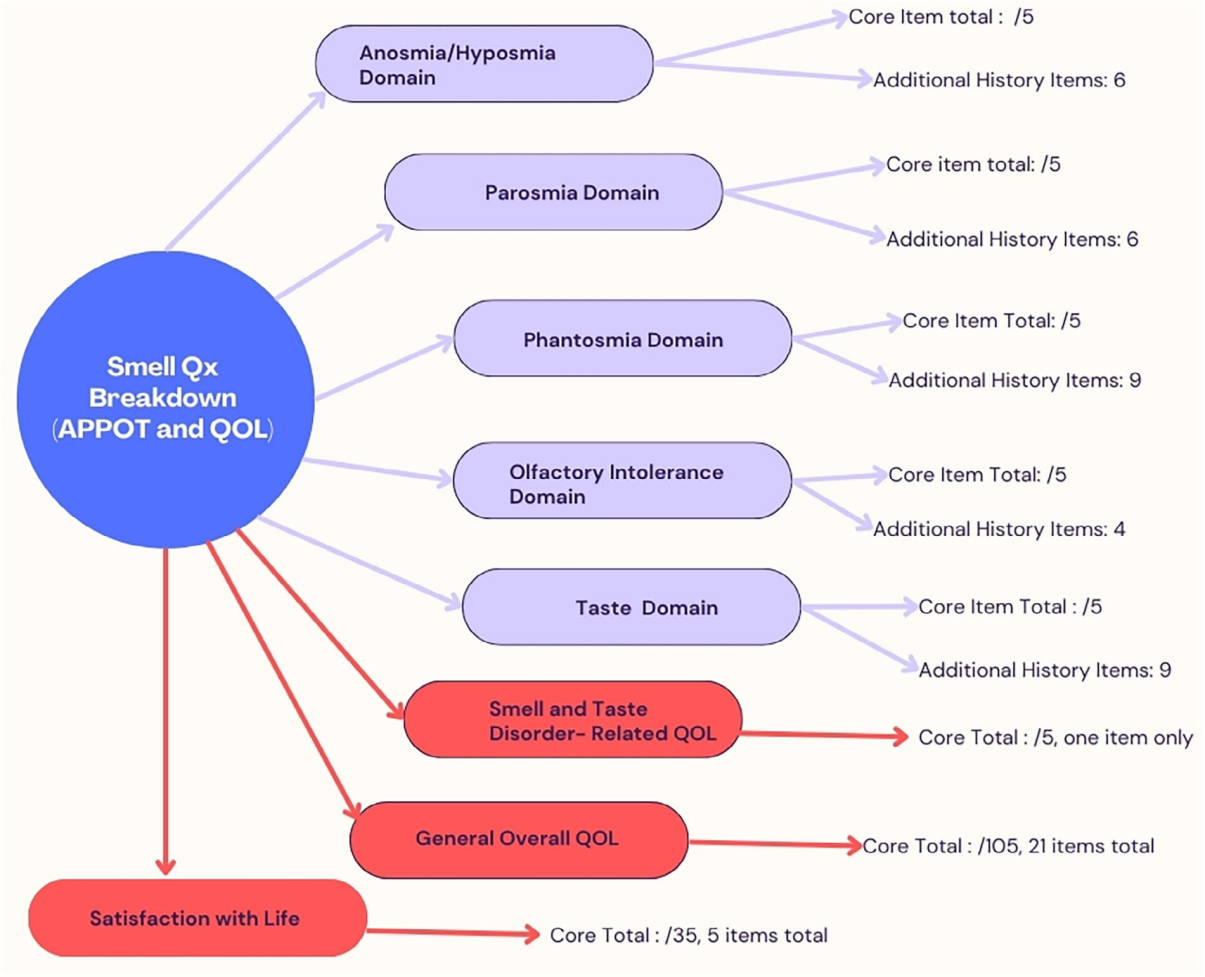
Smell‐Qx breakdown. APPOT breakdown can be seen in purple and QOL and related scores in red.

Finally, satisfaction with life included five items, each scored from 1 to 7, resulting in a total score out of 35 (data analysis questions are highlighted in Supporting Information Appendix 2). The Smell‐Qx also includes history questionnaires, used as part of the initial assessment to gather additional details, such as when the symptoms started, how they have changed over time, and whether they occurred suddenly or developed progressively. The history items do not contribute to the final score but help the physician gain a comprehensive understanding of the patient's olfactory and gustatory symptoms. The breakdown of APPOT and QOL domains can be seen in Figure 1.

### 2.2 Ethical Approval

Ethical approval for clinical validation of this questionnaire was obtained from the Health Research Authority (HRA) Harrow Research Ethics Committee (REC) (REC reference: 24/PR/0099, February 14, 2024), with a non‐substantial amendment (NSA01) for the inclusion of James Paget University Hospitals NHS Foundation Trust, approved on March 27, 2024.

### 2.3 Setting

Patients who presented at the Smell & Taste Clinic at Homerton Healthcare NHS Foundation Trust and James Paget University Hospital with any qualitative or quantitative olfactory dysfunction, or self‐reported gustatory dysfunction (specifically loss of true taste—sweet, salty, sour, bitter, or umami), were recruited for the study. Individuals with a normal TDI score (>30.75) were included if they self‐reported a qualitative smell or taste disorder.

Those presenting to the general Ear, Nose and Throat (ENT) Clinics at these institutions, who did not report any smell or taste problems, were enrolled as control patients. Informed consent was obtained from all participants. The Smell‐Qx takes between 10 and 15 min to complete.

### 2.4 Participants

#### 2.4.1 Inclusion Criteria

Eligible participants were adult patients aged 18 years or older who were able to provide informed consent.

General ENT participants were defined as patients under the care of Adult ENT or a subspecialty clinic other than the Smell and Taste Clinic that did not report any qualitative or quantitative olfactory or self‐reported gustatory dysfunction. Eligible participants in this group were also adult patients aged 18 years or older who were able to provide informed consent.

### 2.5 Variables

All patients recruited to the study also underwent the Sniffin’ Sticks Psychophysical testing as part of the standard‐of‐care to record their TDI scores. Each component of the test is scored out of 16, with a total TDI score of ≥30.75 indicating normosmia [2, 18].

#### 2.5.1 Sample Size Calculation

To determine the appropriate sample size for our study, we performed a Wilcoxon–Mann–Whitney sample size calculation with a two‐sided Type I error rate of 0.05 and a power of 0.8. Using data from the first 10 enrolled patients, we estimated a relative effect size of 0.0423. Based on this effect size, the calculation indicated that seven patients per group would be required to achieve sufficient statistical power. The sample size calculation was conducted using R (Version 4.3.3) with the WMWssp package.

### 2.6 Statistical Analysis

Construct validity was assessed using concurrent and criterion validity. The ability of the Smell‐Qx to reflect “known group” differences was analyzed by assessing the ability of the Smell‐Qx to produce different scores in a group of patients with SATDs versus a control group of patients not known to have SATDs. Concurrent criterion validity examined the Smell‐Qx associations with quality‐of‐life and satisfaction domains and congruent validity was assessed via comparison of the Smell‐Qx total score with the 22‐item Sino‐nasal Outcome Test (SNOT‐22) olfactory item score. Concurrent criterion validity was evaluated further by correlating Smell‐Qx overall and domain scores with Sniffin’ Stick TDI results and individual TDI components.

Data analysis was conducted in IBM SPSS Statistics 27 and GraphPad Prism 10.3.1, with data expressed as mean ± standard deviation. The Shapiro–Wilk test assessed normality, followed by unpaired *t*‐tests for normal data and Mann–Whitney *U* tests for non‐normal data, with *p* < 0.05 considered statistically significant. Pearson correlation coefficients were used to examine relationships across quality‐of‐life domains, SNOT‐22 olfactory item, TDI scores, and Smell‐Qx scores. Reliability was measured through internal consistency (Cronbach's *α* > 0.6) [19] and test–retest reliability, with intraclass correlation coefficient (ICC) assessing stability.

## 3 Results

### 3.1 Systematic Review

The literature search generated 34 results, of which 22 questionnaires or studies were selected which focused on determining questions relevant to olfactory and gustatory dysfunction and were included in the assessment (Figure S1, Table S2) [1, 5, 20, 21, 22, 23, 24, 25, 26, 27, 28, 29, 30, 31, 32, 33, 34, 35, 36, 37, 38, 39]. This pool of items was further sub‐divided based on individual items’ “type”/content. The options for item type were developed to simplify our review of the items and comprised “symptom,” “extent,” “consequence,” “QoL,” and “relevant history.” We then looked at the QoL items more closely and categorized these as a function of their impact (social, eating, anxiety, and annoyance).

Several studies addressed the loss of smell for specific items, including common food items (culture‐specific) and ambient odors, such as cigarette smoke, household gas, flowers, and others [22, 29, 31, 32, 37]. Various qualitative aspects of olfactory dysfunction were assessed across studies, including the extent of dysfunction and the duration and/or frequency of dysfunction. These assessed how patients perceived the severity of their olfactory/gustatory dysfunction [5, 22, 24, 25, 30, 31, 34, 35, 38]. Lastly, most questionnaires assessed the impact of olfactory dysfunction on quality of life, including the impact on taste and eating [5, 22, 24, 30, 32, 35, 36]. Other QoL items included those related to the impact on relationships, the ability to carry out daily activities, and the influence of patients’ emotions and psychological state.

In addition to quantitative and qualitative olfactory symptoms including olfactory loss, parosmia, phantosmia, and olfactory intolerance, other sinonasal symptoms were addressed in relative depth in several questionnaires. Notably, the SNOT‐22 [26], developed as a Patient Reported Outcome Measure (PROM) for rhinosinusitis, covers several domains; however, it includes only a single, generic question on smell and taste without further detail [26, 40]. The DyNaChron questionnaire separates the questions into the domains: nasal obstruction, anterior and posterior rhinorrhea, sense of smell difficulty, facial pain, and cough [32]. Other disease‐specific symptoms include dry mouth, nausea, vomiting, and mouth sores, as addressed by the assessment of taste and smell in cancer patients, developed by Amezaga et al. [35]. The Questionnaire of Olfactory Disorders (QoD) briefly queries the presence of nasal and oral symptoms [24].

Altogether, this review yielded a list of items related to olfactory and gustatory dysfunction, which were then used for the development of Smell‐Qx.

### 3.2 Face and Content Validity

Following item generation and selection, face and content validity were confirmed through a Delphi process with international experts in SATDs, who assessed each item's relevance, clarity, and essential symptom coverage, resulting in 11 domains (see Section 2 for details).

The Smell‐Qx questionnaire consists of 11 domains addressing SATDs, including olfactory loss, parosmia, phantosmia, olfactory intolerance, gustation, symptom descriptors (e.g., laterality), etiology, trigeminal symptoms, quality of life, and life satisfaction, totaling 70 items. Each core symptom domain begins with a screening question, followed by a five‐point Likert scale rating (1 = very mild to 5 = worst possible problem) for severity.

Domains feature varied question types—Likert scales, rating scales, and yes/no—to capture symptom breadth and impact depth.

The parosmia domain includes a scale from −5 (most disgusting) to 5 (most pleasant) for different odors, including pleasant (e.g., chocolate), neutral (e.g., coffee), and unpleasant (e.g., garlic) stimuli.

Following the Delphi and consultation phases, domains 1–5 (Olfactory Dysfunction, Parosmia, Phantosmia, Olfactory Intolerance, and Taste Disturbance) were designated as core domains for overall score calculation, while domains 5–11 were categorized as quality‐of‐life and history domains to offer a deeper understanding of daily life impact and additional clinical insights.

### 3.3 Participants

The average age of the respondents was 45.8 years, ranging from 20 to 88 years. A total of 60 participants were recruited (32 patients with smell and/or taste dysfunction and 28 controls), consisting of 35 males and 24 females, with one individual opting not to disclose their gender.

Overall, the etiology of SATDs included post‐infectious olfactory dysfunction (*n* = 16, 50.0%), idiopathic (*n* = 8, 25.0%), chronic rhinosinusitis with nasal polyps (*n* = 3, 9.4%), iatrogenic (drug exposure) (*n* = 2, 6.3%), toxic rhinitis (chemical irritant exposure) (*n* = 1, 3.1%), post‐sinus surgery (*n* = 1, 3.1%), and post‐traumatic olfactory dysfunction (*n* = 1, 3.1%).

Moreover, TDI testing demonstrated 53.1% (*n* = 17) of patients with hyposmia (score = 16.5–30.75), 31.3% (*n* = 10) with anosmia (score < 16.5) and 15.6% (*n* = 5) tested within the normosmia range (score >30.75). Those within the normosmia range all (*n* = 5) reported qualitative olfactory disorders. The mean SNOT‐22 score was significantly different between control patients and patients with SATDs (mean_control_ = 17.5, 95% CI: 10.59–25.66, vs. mean_test_ = 38.25, 95% CI: 29.30–47.20, *p* = 0.0003). Furthermore, the mean SNOT‐22 olfactory item score was significantly higher in patients with SATD's (mean_test_ = 2.28, 95% CI: 1.85–2.71) versus control (mean control = 0), *p* < 0.001.

### 3.4 Outcome Data

#### 3.4.1 Internal Consistency

The internal consistency of the different domains for patients with SATDs (*n* = 32) is shown in Table 1. The Quality of Life and Satisfaction with Life items demonstrated excellent internal consistency (*α* = 0.95), as did the Parosmia domain (*α* = 0.97) and the Taste domain (*α* = 0.87). Both the smell loss domain (*α* = 0.77) and the Phantosmia domain (*α* = 0.64) demonstrated acceptable internal consistency. The olfactory intolerance domain had only one Likert item and so internal consistency was not calculated.

**TABLE 1:** Internal consistency of domains.

	Cronbach's *α*
Smell loss domain	0.77 (2 items)
Parosmia domain	0.97 (9 items)
Phantosmia domain	0.64 (2 items)
Taste domain	0.87 (6 items)
SATD‐related quality of life, general quality of life, and satisfaction with life	0.95 (27 items)

#### 3.4.2 Test–Retest

The ICC was used to evaluate the test‐retest reliability of the overall Smell‐Qx score. Seventeen patients with SATDs completed the Smell‐Qx survey at two time points, with a mean interval of 15.2 days between assessments (range: 7–24 days). The analysis showed excellent reliability, with an ICC of 0.95 for single measures and 0.97 for average measures (both *p* < 0.001). The single and average ICC measures for the overall‐SATD‐related QOL (0.65, 0.79), overall general QOL (0.99, 0.99), and satisfaction with life (0.90, 0.95) can be seen in Table 2. The mean number of days between responses was 13.4 (95% CI: 11.36–15.44).

**TABLE 2:** Intraclass correlation coefficients (ICCs) for the test–retest reliability of the questionnaire domains. The ICC values for single and average measures, along with their 95% confidence intervals (CI), F‐values, degrees of freedom (df), and significance (Sig) levels, are reported.

		Intraclass Correlation	95% Confidence Interval Lower Bound	Upper Bound	F Test with True Value 0 (n = 17) Value	df1	df2	Sig
Overall APPOT score	Single Measures	0.95	0.86	0.98	35.441	16	16	<0.0001
	Average Measures	0.97	0.922	0.99	35.441	16	16	<0.0001
SATD‐related Qol	Single Measures	0.65	0.27	0.86	4.761	16	16	0.002
	Average Measures	0.79	0.42	0.92	4.761	16	16	0.002
Overall General QOL	Single Measures	0.99	0.98	0.99	318.589	16	16	<0.0001
	Average Measures	0.99	0.99	0.99	318.589	16	16	<0.0001
Satisfaction with Life	Single Measures	0.90	0.74	0.96	18.765	16	16	<0.0001
	Average Measures	0.95	0.85	0.98	18.765	16	16	<0.0001

Abbreviations: APPOT score, Anosmia/Hyposmia, Parosmia, Phantosmia, Olfactory Intolerance, Taste score; SATD, Smell and Taste Disorders; QOL, Quality of Life.

### 3.5 Types of Olfactory and Gustatory Dysfunction

Of the 32 patients with smell and/or taste disorders, 21.9% (*n* = 7) reported symptoms in one dysosmia/taste domain, 21.9% (*n* = 7) reported symptoms in two domains, 15.6% (*n* = 5) reported symptoms in three, 31.3% (*n* = 10) in four, and 9.4% (*n* = 3) in five domains. Additionally, 53.1% (*n* = 17) of those reporting smell loss also experienced parosmia, and 62.5% (*n* = 20) of those with smell loss also self‐reported taste loss. Note that 90.1% (*n* = 29) reported at least one qualitative olfactory disorder with only three patients reporting no qualitative disorders.

Table 3 summarizes the distribution of patients reporting smell loss, parosmia, phantosmia, olfactory intolerance, or self‐reported taste loss. Of the five patients, who were normosmic on Sniffin’ Sticks testing (TDI > 30.75), one reported phantosmia without smell loss. The other four patients had a mean score of 3.7 (95% CI: 3.53–3.87) in the smell loss domain, indicating moderate to severe subjective loss despite normal objective function. Among these, two also reported phantosmia, one had parosmia, and one experienced gustatory dysfunction.

**TABLE 3:** Breakdown by domain. Significant differences were observed in TDI scores between individuals with and without phantosmia (24.1 vs. 18.2, *p* = 0.0305). A significant difference in overall APPOT scores was found between those with and without smell loss (9.6 vs. 3.0, *p* = 0.0262), those with and without parosmia (11.7 vs. 6.4, *p* = 0.0012), and those with and without olfactory intolerance (14.3 vs. 7.2, *p* < 0.001). Individuals with olfactory intolerance also had significantly lower SATD‐related QOL scores (4.1 vs. 2.8, *p* = 0.0017) and overall general QOL scores (57.8 vs. 22.2, *p* = 0.003) compared to those without olfactory intolerance.

	Number of patients affected	Mean duration of symptoms (months)	Average smell TDI (mean)	Overall APPOT score (mean)	SATD‐related QOL overall	Overall general QOL (mean)	Satisfaction with life (mean)
Smell loss/anosmia/hyposmia	*N* = 30	62.4 (6–528)	20.3	9.6	3.2	32.8	19.1
Parosmia	*N* = 17	73.5 (8–528)	19.8	11.65	3.2	32.1	16.7
Phantosmia	*N* = 14	35.4 (8–144)	24.1	10.6	3.1	29.21	19.00
Olfactory intolerance	*N* = 9	93.2 (10–528)	21.1	14.3	4.1	57.8	15.8
Self‐reported taste loss	*N* = 21	59.7 (7–528)	20.3	10.3	3.2	35.6	18.6

Abbreviations: APPOT score, Anosmia/Hyposmia, Parosmia, Phantosmia, Olfactory Intolerance, Taste score; SATD, smell and taste disorders; QOL, quality of life.

Differences in smell loss, parosmia, phantosmia, olfactory intolerance, and taste domains, along with overall APPOT scores and related quality‐of‐life metrics, are presented in Table 4. Individuals with olfactory intolerance had significantly worse SATD‐related QOL scores (mean: 4.1 vs. 2.8, *p* = 0.0017) and overall general QOL total scores (mean: 57.8 vs. 22.2, *p* = 0.0003) compared to those without olfactory intolerance. These differences were not observed for other quantitative or qualitative disorders.

**TABLE 4:** Validation of Smell‐Qx five major domains in SATD cohort versus controls. Criterion and concurrent validity were assessed among patients with SATDs only.

Comparison	Result	*p* value
**Known group differences**		
APPOT SATD vs. control	*t* = 9.99, df = 58	**<0.0001**
**Criterion validity**		
APPOT vs. smell TDI	*r* = −0.11 CI: −0.44 to 0.25	0.5546
APPOT vs. threshold	*r* = −0.17 CI: −0.49 to 0.19	0.3626
APPOT vs. discrimination	*r* = −0.46 CI: −0.70 to −0.14	0.0078
APPOT vs. identification	*r* = −0.01 CI: −0.36 to 0.34	0.9412
Smell loss/anosmia/hyposmia domain vs. smell TDI	*r* = −0.54 CI: −0.79 to −0.15	**0.0108**
Smell loss domain vs. threshold	*r* = −0.35 CI: −0.63 to −0.0070	**0.0463**
Smell loss domain vs. discrimination	*r* = −0.46 CI: −0.70 to −0.14	**0.0078**
Smell loss domain vs. identification	*r* = −0.40 CI: −0.66 to −0.047	**0.0240**
**Concurrent validity**		
SATD‐related quality of life domain and APPOT overall	*r* = 0.43 CI: 0.093 to 0.68	**0.0150**
Overall general QOL and APPOT	*r* = 0.37 CI: 0.0060 to 0.65	**0.0410**
Satisfaction with life domains and APPOT	*r* = −0.31 CI: −0.60 to 0.051	0.0906
Olfactory domain of SNOT22 vs. APPOT	*r* = 0.40 CI: 0.061 to 0.66	**0.0228**

Abbreviations: APPOT score, Anosmia/Hyposmia, Parosmia, Phantosmia, Olfactory Intolerance, Taste score. SATD, smell and taste disorders; QOL, quality of life.

Bold values indicate statistical significance at p < 0.05.

#### 3.5.1 Known Group Differences

**FIGURE 2:**
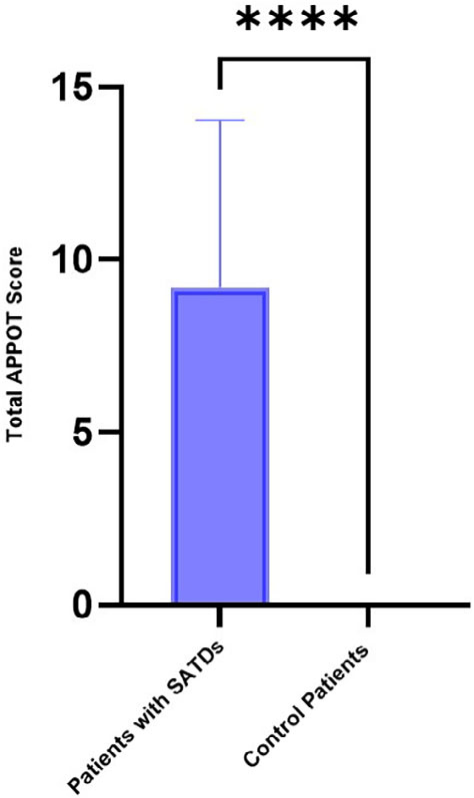
Smell‐Qx overall scores in smell patients with loss of smell versus controls.

The mean overall APPOT score for patients with SATDs was 9.2 (95% CI: 7.43–10.97), compared to 0 in control (*p* < 0.0001) (Figure 2). The Smell‐Qx was able to differentiate between patients with subjective SATDs and control patients (*t* = 9.99, df = 58, *p* < 0.0001). Moreover, patients with SATD had significantly worse overall general QOL scores (32.2 ± 26.2) versus control patients (1.1 ± 3.8) (*p* < 0.001). Similarly, SATD patients had significantly worse satisfaction with life scores (19.3 ± 8.2) versus control patients (29.3 ± 5.8) (*p* < 0.001) (Figures S2 and S3).

#### 3.5.2 Concurrent and Congruent Validity

Concurrent criterion validity was assessed by comparing the Smell‐Qx functional scores with QOL and satisfaction with life scores. A significant correlation was found between the overall APPOT score and the overall SATD‐related QOL domain (*r* = 0.43, *p* = 0.0150, 95% CI: 0.093–0.68). Similarly, a significant correlation was observed between the APPOT score and the general QOL total relating specifically to social life, relationships, food, emotions, hygiene, and danger (*r* = 0.37, *p* = 0.0410, 95% CI: 0.0060–0.65). However, no significant correlation was found between the APPOT score and satisfaction with life scores (*r* = ‐0.31, *p* = 0.0906, 95% CI: −0.60 to 0.051) (Table 4).

Congruent validity was assessed by comparing the APPOT total score with the SNOT‐22 olfactory item, which showed a significant correlation (*r* = 0.82, *p* < 0.0001, 95% CI: 0.67–0.91).

Criterion validity was also assessed by comparing the APPOT scores with patients' overall Smell TDI scores. No correlation was observed between the overall TDI scores (*r* = −0.11, *p* = 0.5546, 95% CI: −0.44 to 0.25) or the individual threshold (*r* = −0.17, *p* = 0.3626, 95% CI: −0.49 to 0.19), discrimination (*r* = −0.10, *p* = 0.5901, 95% CI: −0.43 to 0.26), and identification (*r* = −0.01, *p* = 0.9412, 95% CI: −0.36 to 0.34) results with the APPOT overall scores.

**FIGURE 3:**
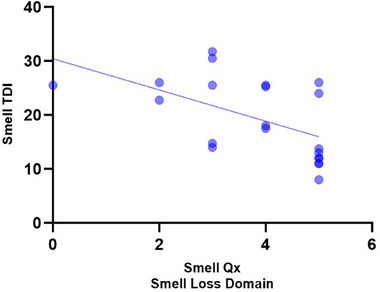
Correlation of Smell‐Qx smell loss domain with overall smell TDI.

However, a correlation was observed when comparing the Smell‐Qx smell loss domain scores with overall Smell TDI (*r* = −0.54, *p* = 0.0108, 95% CI: −0.79 to −0.15) (Figure 3). Correlations were also observed between the threshold (*r* = −0.35, *p* = 0.0463, 95% CI: −0.63 to −0.0070), discrimination (*r* = −0.46, *p* = 0.0078, 95% CI: −0.70 to ‐0.14), and identification (*r* = −0.40, *p* = 0.0240, 95% CI: −0.66 to −0.047) scores (Table 4).

## 4 Discussion

### 4.1 Key Results

In this study, we have developed and validated the Smell‐Qx questionnaire to assess patients with olfactory and taste dysfunction and its impact on quality of life. Our results indicate that the Smell‐Qx is a valid tool for assessing patient‐reported outcomes in this population, demonstrating internal consistency, test–retest reliability, and validity. The Smell‐Qx is also able to determine differences in QOL metrics between SATD patients and controls, suggesting it can be used as a tool for assessing both symptom severity and the impact on quality of life.

### 4.2 Limitations

The study has some limitations. First, the test–retest statistics may have been affected by response bias, as not all patients completed assessments within the same timeframe, a common limitation in questionnaire validation. Comparisons between patients with parosmia, phantosmia, or olfactory intolerance were limited, as many experienced more than one type of olfactory dysfunction. Comparing quality‐of‐life metrics between those with and without qualitative disorders was also restricted. Psychophysical taste function was not measured, limiting the assessment of the gustatory domain's concurrent validity. Control patients did not undergo psychophysical smell or taste testing, making it hard to exclude undiagnosed disorders in this group. The etiologies and their incidences in this study may not reflect the broader SATD patient population.

### 4.3 Interpretation

The study combined psychosocial measures of olfaction, including the Sniffin’ Sticks TDI and Quality of Life comparators. The Smell TDI did not correlate with the APPOT score, supporting prior findings that the TDI and PROMs assess different olfactory aspects [1]. Disparities between subjective and objective olfactory dysfunction have been noted, highlighting challenges in linking self‐reported smell with impacts on daily life and quality of life [1, 3, 4]. For instance, patients with hyposmia may report normal smell, while normosmic individuals might report poor smell [3, 5, 38, 41, 42, 43]. Here, four patients reported moderate to severe smell loss despite normal TDI. Further analysis found a significant negative correlation between the Smell‐Qx smell loss domain and TDI scores, establishing a link with psychophysical testing. Differentiating “measured” from “subjective” olfactory function enables patient‐centered care [2].

The Smell‐Qx uniquely includes chemosensory dysfunction items, particularly for parosmia, along with symptom frequency and progression—features absent in other questionnaires [31, 32, 37, 38]. While the TASTE questionnaire covers smell and taste loss, it has limited questions on qualitative disorders [38]. Similarly, ODOR addresses smell quality of life but lacks qualitative disorder detail [36].

In our cohort, 78.2% reported two or more simultaneous smell or taste disorders. Although ODOR, TASTE, ASOF, and DyNaChron capture quality‐of‐life metrics for smell loss, they do not address the full spectrum of dysfunctions [31, 32, 38, 40]. While the USA Smell and Taste Patient Survey and Questionnaire for Olfactory Disorders include qualitative disorder aspects, they use presence/absence questions rather than Likert scales for severity [1, 24, 35].

### 4.4 Generalizability

The Smell‐Qx, therefore, not only provides a method for measuring quantitative smell loss—shown to correlate with the Smell TDI—but also offers an alternative approach for identifying patients with qualitative disorders. Crucially, qualitative disorders, such as parosmia, phantosmia, and olfactory intolerance, are often not detected by commonly used psychophysical tests like the Sniffin' Sticks TDI or the SIT40 [2, 3]. While the Sniffin' Sticks Parosmia Test (SSParoT) has been tried to assess parosmia and track changes in the condition, it has not yet proven its worth in parosmia; furthermore, other qualitative olfactory disorders remain a challenge [44, 45]. The Smell‐Qx offers a novel approach for monitoring qualitative symptoms over time, as well as assessing the impact on quality of life in response to treatment. This makes it an effective tool for tracking both symptom progression and the broader effects on patients well‐being.

## 5 Conclusion

The Smell‐Qx is a reliable and valid PROM for assessing SATDs, providing an effective tool for screening, monitoring symptoms, and evaluating their impact on quality of life. Its smell domain correlates with psychophysical olfactory tests, though the overall score does not. This suggests the Smell‐Qx can complement psychophysical testing in a multi‐modal approach to assess smell and taste disorders.

## Author Contributions

*Conceptualization*: Matt Lechner, Alexander Fjaeldstad, Carl Philpott, and Thomas Hummel. *Methodology*: Matt Lechner, Alexander Fjaeldstad, Umar Rehman, Jacklyn Liu, David Boniface, Jim Boardman, Duncan Boak, Aytug Altundag, Johannes Frasnelli, Simon Gane, Eric Holbrook, Julien Hsieh, Caroline Huart, Iordanis Konstantinidis, Basile N. Landis, Valerie J. Lund, Alberto Macchi, Eri Mori, Christian Mueller, Joaquim Mullol, Simona Negoias, Zara M. Patel, Jayant M. Pinto, Sophia Poletti, Vijay Ramakrishnan, Philippe Rombaux, Jan Vodicka, Antje Welge‐Luessen, Katherine L. Whitcroft, Carol Yan, Carl Philpott, and Thomas Hummel. *Data acquisition*: Matt Lechner, Alexander Fjaeldstad, Carl Philpott, Thomas Hummel, Umar Rehman, and Jacklyn Liu. *Formal analysis and investigation*: Matt Lechner, Alexander Fjaeldstad, Umar Rehman, Jacklyn Liu, David Boniface, Jim Boardman, Duncan Boak, Aytug Altundag, Johannes Frasnelli, Simon Gane, Eric Holbrook, Julien Hsieh, Caroline Huart, Iordanis Konstantinidis, Basile N. Landis, Valerie J. Lund, Alberto Macchi, Eri Mori, Christian Mueller, Joaquim Mullol, Simona Negoias, Zara M. Patel, Jayant M. Pinto, Sophia Poletti, Vijay Ramakrishnan, Philippe Rombaux, Jan Vodicka, Antje Welge‐Luessen, Katherine L. Whitcroft, Carol Yan, Carl Philpott, and Thomas Hummel. *Writing – original draft preparation*: Matt Lechner, Alexander Fjaeldstad, Carl Philpott, Thomas Hummel, Umar Rehman, and Jacklyn Liu. *Supervision*: Matt Lechner, Alexander Fjaeldstad, Carl Philpott, and Thomas Hummel.

## Conflicts of Interest

C.P. is Director of Research and Medical Affairs on the board of trustees for the UK Charity Fifth Sense. D.B. is the CEO of Fifth Sense. J.B. is the head of the PPI Panel for Fifth Sense. The remaining authors have no conflicts of interest.

## Supporting information



Supporting Information

Supporting Information

## Data Availability

The data that support the findings of this study are available from the corresponding author upon reasonable request.
