# Supplementary material for: The Development and Validation of the Smell‐Qx Questionnaire, Based on a Systematic Review of the Literature and the COMET Initiative on the Development of Core Outcome Sets for Clinical Trials in Olfactory Disorders
Source: Int Forum Allergy Rhinol. 2025 May 9;15(9):974–84. doi: 10.1002/alr.23604 (PMC12401079; doi:10.1002/alr.23604)
Supplement: Supplementary file 1 — Supporting Information [file ALR-15-974-s001.docx]

**Appendix 1**

| **Date** | **Database** | **Search Terms** |
| --- | --- | --- |
| May 2023 | Pubmed | ((((((((olfactory disorder OR anosmia) OR hyposmia) OR loss of smell) OR smell) OR olfact*) OR cacosmia) OR dysosmia) AND (surveys and questionnaires) AND (reproducibility of results) |

**Supplemental Table 1. Search Terms.**

|  | **Reference** | **Description** |
| --- | --- | --- |
| 1 | Gent et al, 1987.^19^ | Taste and smell problems: Validation of questions for the clinical history |
| 2 | Nordin et al, 1996.^20^ | Assessment of qualitative olfactory dysfunction |
| 3 | Nordin et al, 2003.^21^ | Scandinavian adaptation of the multi-clinic smell and taste questionnaire (MCSTQ-Sc) |
| 4 | Olsson et al, 2003.^22^ | Self-reported allergic and non-allergic rhinitis symptoms |
| 5 | Frasnelli and Hummel, 2005.^23^ | Questionnaire of Olfactory Disorders (QOD) |
| 6 | Goldberg et al, 2005.^24^ | ChemoSensory Questionnaire for head and neck cancer patients |
| 7 | Hopkins et al, 2009.^25^ | 22-Item Sinonasal Outcomes Test (SNOT-22) |
| 8 | Croy et al, 2010.^26^ | Individual significance of olfaction |
| 9 | Landis et al, 2010.^27^ | Clinical usefulness of structured questions in parosmia |
| 10 | Takebayashi et al, 2011.^28^ | Self-administered odor questionnaire |
| 11 | Wehling et al, 2011.^29^ | Subjectively assessed olfactory function |
| 12 | Pusswald et al, 2012.^30^ | 12 Item validated questionnaire for the assessment of self-reported olfactory functioning and olfaction-related quality of life (ASOF) |
| 13 | Kacha et al, 2012.^31^ | Dysfonctionnement Nasal Chronique (DyNaChron) |
| 14 | Mullol et al, 2012.^5^ | OLFACAT (olfaction in Catalonia) |
| 15 | Simopoulos et al, 2012.^32^ | Olfaction-associated quality of life in chronic rhinosinusitis |
| 16 | Balaguer, Percodani and Woisard, 2017.^33^ | The Carcinologic Handicap Index |
| 17 | Amezaga et al, 2018.^34^ | Taste and smell alterations in cancer patients |
| 19 | Mattos et al, 2019.^35^ | A brief version of the questionnaire of olfactory disorders in patients with chronic rhinosinusitis |
| 20 | Langstaff et al, 2019.^1^ | Anglised version of the Questionnaire of Olfactory Disorders (eODQ) |
| 21 | Lee et al, 2022.^36^ | Olfactory Dysfunction Outcomes Rating |
| 22 | Niklassen et al 2022.^37^ | Taste and Smell Tool for Evaluation (TASTE) questionnaire |
| 18 | Naimi et al 2023.^38^ | USA Smell and Taste Patient Survey |

**Supplemental Table 2.** Questionnaires/studies measuring changes related to olfactory and gustatory symptoms.

**Identification of studies via databases and registers**

Records identified from:

Ovid MEDLINE(R) 1946 to 2023

1946-2023

**Identification**

Records screened

(n = 34)

Records excluded

(n = 0)

Reports sought for retrieval

(n = 34)

Reports not retrieved

(n = 0)

**Screening**

Reports assessed for eligibility

(n = 34)

Reports excluded:

Did not include validation/reliability analysis of a novel or modified PROM (n=12)

Studies included in review

(n = 22)

**Included**

**Supplemental Figure 1.** PRISMA flow diagram of systematic literature search.

**Supplemental Figure 2.** Total General QOL Scores SATD Patients Versus Control

**Supplemental Figure 3.** Total Satisfaction with Life Scores SATD Patients Versus Control.
