# Supplementary material for: The Development and Validation of the Smell‐Qx Questionnaire, Based on a Systematic Review of the Literature and the COMET Initiative on the Development of Core Outcome Sets for Clinical Trials in Olfactory Disorders
Source: Int Forum Allergy Rhinol. 2025 May 9;15(9):974–84. doi: 10.1002/alr.23604 (PMC12401079; doi:10.1002/alr.23604)
Supplement: Supplementary file 2 — Supporting Information [file ALR-15-974-s002.docx]

**Appendix 2. Smell Qx.** Items highlighted in yellow were used to calculate the overall Smell Qx symptom/sensory score. Those highlighted in purple contribute to the overall quality of life score, while blue-highlighted items contribute to quality-of-life subdomains, including social relationships, food, emotions, and hygiene. The remaining items are part of the history screening questions, helping to provide a comprehensive overview of the patient's olfactory and gustatory symptoms.

**Baseline Demographics**

1. What gender do you identify with?
2. Male b. Female c. Non-binary d. Transgender male e. Transgender female d. Other (please specify)…………….
3. How old are you (Please specify in years)

………………….

1. What is your relationship status?
2. Single b. Married/Civil Partnership c. Partnered d. Separated/Divorced e. Widowed f. Other (Please Specify) ……………
3. What is your ethnic origin/ancestry
4. White/Caucasian b. Mixed Race c. Asian/Asian British d. Black/Black British e. Chinese or Chinese British f. Other (Please Specify) …. f. Prefer not to specify.
5. Please state which city you currently live in?

……………...

**Date of assessment:**

**1) Olfactory Dysfunction DOMAIN**

**1) Is your sense of smell reduced (weakened) or completely absent?**

☐ Yes [-> if yes -> 1a, 1b, etc.] ☐ No [-> ‘no problem’ -> continue with 2]

**1a) My sense of smell is**

☐ reduced (weakened), but not completely lost

☐ completely absent/lost (I do not smell anything)

**1b) How would you rate this problem?**

1 – very mild problem

2 – mild or slight problem

3 – moderate problem

4 – severe problem

5 – problem as bad as it can be

**1c) When did this problem start? If you are not certain, please give your best estimate.**

☐ MM/YYYY

**1d) Did this problem occur:**

☐ Suddenly

☐ Gradually -> if ticked -> then

☐Over a period of __XX___ days

☐ Over a period of __XX___months

☐ Over a period of __XX___years

☐ I am not sure

☐ I had this problem since I was born

**1e) Please indicate which box applies to how your smell problem (reduced/weakened smell or absent smell/smell loss) changed since the time of onset:**

|  | Unchanged | Improved | Worsened |  |
| --- | --- | --- | --- | --- |
|  | ☐ | ☐ | ☐ |  |

**1f) Please indicate which box applies to how your smell problem (reduced/weakened smell or absent smell/smell loss) changed over the last three months (please answer if the problem persisted for more than 3 months):**

|  | Unchanged | Improved | Worsened |  |
| --- | --- | --- | --- | --- |
|  | ☐ | ☐ | ☐ |  |

**1g) How would you rate your sense of smell today (0 being completely absent/no sense of smell and 10 being completely normal)?**

0 (completely absent/no sense of smell) ------------------10 (completely normal)

**2) PAROSMIA DOMAIN**

**2)** **Have you noticed that smell(s) smell differently (distorted) than they used to?**

☐ Yes [-> 2a, 2b, etc.] ☐ No [-> ‘no problem’ -> continue with 3]

**2a) How would you rate this problem?**

1 – very mild problem

2 – mild or slight problem

3 – moderate problem

4 – severe problem

5 – problem as bad as it can be

**2b) When did this problem start? If you are not certain, please give your best estimate.**

☐ MM/YYYY

**2c) Please indicate which box applies to how your smell problem (my smell is distorted, meaning things smell differently than they used to) changed since the time of onset:**

|  | Unchanged | Improved | Worsened |  |  |
| --- | --- | --- | --- | --- | --- |
|  | ☐ | ☐ | ☐ |  |  |

**2d) Please indicate which box applies to how your smell problem (my smell is distorted, meaning things smell differently than they used to) changed over the last three months (please answer if the problem persisted for more than 3 months):**

|  | Unchanged | Improved | Worsened |  |  |
| --- | --- | --- | --- | --- | --- |
|  | ☐ | ☐ | ☐ |  |  |

**2e) When you clamp or pinch your nose do you still perceive the distorted smell?**

☐ Yes ☐ No

**2f) How would you describe the quality of most smells that are distorted? (please circle most appropriate response)**

-5 (most disgusting),-4, -3, -2, -1, 0, 1, 2, 3, 4, 5 (most pleasant)

**2g) How do you perceive the following smells? (please circle response)**

Coffee

-5 (most disgusting),-4, -3, -2, -1, 0, 1, 2, 3, 4, 5 (most pleasant)

Egg

-5 (most disgusting),-4, -3, -2, -1, 0, 1, 2, 3, 4, 5 (most pleasant)

Onion

-5 (most disgusting),-4, -3, -2, -1, 0, 1, 2, 3, 4, 5 (most pleasant)

Mint/Toothpaste

-5 (most disgusting),-4, -3, -2, -1, 0, 1, 2, 3, 4, 5 (most pleasant)

Roasted meat

-5 (most disgusting),-4, -3, -2, -1, 0, 1, 2, 3, 4, 5 (most pleasant)

Garlic

-5 (most disgusting), -4, -3, -2, -1, 0, 1, 2, 3, 4, 5 (most pleasant)

Chocolate

-5 (most disgusting),-4, -3, -2, -1, 0, 1, 2, 3, 4, 5 (most pleasant)

Petrol/oil

-5 (most disgusting), -4, -3, -2, -1, 0, 1, 2, 3, 4, 5 (most pleasant)

2h) How many smells do you perceive as distorted?

☐ none ☐ few ☐ many ☐ most ☐ all

**3) PHANTOSMIA DOMAIN**

**3)** **Have you noticed a smell when no source of smells is present and no one else can smell them (phantom smell)?**

☐ Yes (-> 3a, 3b, etc.) ☐ No (-> ‘no problem’ -> continue with 4)

**3a) How would you rate this problem?**

1 – very mild problem

2 – mild or slight problem

3 – moderate problem

4 – severe problem

5 – problem as bad as it can be

**3b)** **When did this problem start? If you are not certain, please give your best estimate.**

☐ MM/YYYY

**3d) Please indicate which box applies to how your smell problem (phantom smell(s), meaning I notice smells when no source of smells is present and no one else can smells them) changed since the time of onset:**

|  | Unchanged | Improved | Worsened |  |
| --- | --- | --- | --- | --- |
|  | ☐ | ☐ | ☐ |  |

**3d) Please indicate which box applies to how your smell problem (phantom smell(s), meaning I notice a smell when no source of smell is present and no one else can smell them) changed over the last three months (please answer if the problem persisted for more than 3 months):**

|  | Unchanged | Improved | Worsened |  |
| --- | --- | --- | --- | --- |
|  | ☐ | ☐ | ☐ |  |

**3e) Does anyone near to you notice the smell too?**

☐ Yes ☐ No

**3f) How would you describe the quality of the phantom smell that you experience?**

-5 (most disgusting),-4, -3, -2, -1, 0, 1, 2, 3, 4, 5 (most pleasant)

Can you describe the perceived odour(s)/smell(s)?

………………..

**3g) How often do you experience the phantom smell?**

☐ every day

☐ XX days per week

☐ XX days per month

**3h) How long do these phantom smells last for?**

☐ Seconds

☐ Minutes

☐ hours

☐ days

☐ all the time

**3i) Is there a trigger for these phantom smells?**

☐ Yes ☐ No

-> If no -> Is it there all the time? ☐ Yes ☐ No

-> If yes -> What triggers the phantom smell to come on?

□ certain smells (odours)

(if ticked ->)

What smells?

……………………………….

□ burning/stinging/cooling smells

□ air flow (feeling of air flowing through your nose)

□ other

……

**3j) Can you stop these smells by using any of the following manoeuvres?**

☐ Blocking the nose, e.g. by clamping or pinching

nose

☐ Being around certain smells

☐ Valsalva maneuver (when you are on an aircraft or diving and equalize the pressures in your ears by pinching your nose and trying to breathe out).

☐ Change of head position

☐ Sniffing in hard

☐ Other -> please specify …

☐ I cannot stop these smells by using any of the above manoeuvres

**4) DOMAIN vs. HYPERSENSITIVITY DOMAIN**

**OLFACTORY INTOLERANCE**

**4) Have you noticed that your sense of smell is more sensitive (to an extent that you do not tolerate certain smells), but the smells themselves are not distorted?**

☐ Yes (-> 4a, 4b, etc.) ☐ No (-> ‘no problem’ [according to SNOT-22 scale] -> continue with 5)

**4a) How would you rate this problem?** [according to SNOT-22 scale]

1 – very mild problem

2 – mild or slight problem

3 – moderate problem

4 – severe problem

5 – problem as bad as it can be

**4b) When did this problem start? If you are not certain, please give your best estimate.**

☐ MM/YYYY

**4c) Please indicate which box applies to how your smell problem (more sensitive smell) changed since the time of onset:**

|  | Unchanged | Improved | Worsened |  |
| --- | --- | --- | --- | --- |
|  | ☐ | ☐ | ☐ |  |
|  |  |  |  |  |

**4d) Please indicate which box applies to how your smell problem (more sensitive smell) changed over the last three months (please answer if the problem persisted for more than 3 months):**

|  | Unchanged | Improved | Worsened |  |
| --- | --- | --- | --- | --- |
|  | ☐ | ☐ | ☐ |  |

**4e) How often do you experience this problem?**

☐ every day

☐ XX days per week

☐ XX days per month

**5) to 7) - GENERAL SMELL QUESTIONS (if the patient ticked ‘yes’ in any of question 1-4)**

**5) The smell problem affects**

☐ One side -> if yes -> ☐ right side ☐ left side

☐ Both sides

☐ I don’t know

**6) Does your smell problem fluctuate, meaning that it gets better sometimes, then worse again?**

☐ Yes

☐ No

**7) Did your smell problem begin when you had (check all that apply) [? LINK EARLIER IF PATIENT ANSWERS YES TO ANY OF THE ABOVE DOMAINS’:**

☐ Acute upper respiratory infection (e.g. common cold, **COVID-19** or flu, other infection) – please specify………….

☐ Nasal disease (e.g. problems breathing through the nose, hay fever, sinusitis, polyps,) – If yes, please specify: ______________________

☐ Accident/head/nose injury – If yes, please specify: ______________________

☐ I was born this way because I have no memories of having ever smelled or tasted anything in the past

☐ Problems with my teeth (dental problems)/dentures – If yes, please specify: _________________ and did you see a dentist during the last year? ☐ Yes ☐ No

☐ There has been a change in my work place or home environment (exposed to chemicals (eg. herbicides, pesticides, chemicals, smoke, heavy metals, such as Cadmium or Chrome)

If yes, please specify: ______________________

☐ X-ray therapy (radiation) or chemotherapy for cancer– If yes, please specify: _______________________________________________

☐ Dry mouth

☐ Chronic kidney disease

☐ Other illness, stroke – If yes, please specify: ______________________

☐ Surgery/general anaesthetic (both nose/throat surgery, neurosurgery and other surgery)

– If yes, please specify: ______________________

☐ It started with a new medication (specifically amoxicillin/azithromycin/ciprofloxacin/fluticasone/prednisolone/amlodipine/diltiazem/enalapril/atorvastatin/lovastatin/pravastatin/levothyroxine)

- if yes, please specify: ______________________

☐ It started with recreational drug use (cocaine, amphetamine, Crystal etc.)

☐ It started with migraines/I usually get it before I get a headache

☐ It started during pregnancy [FEMALE PATIENTS ONLY – permutation to be programmed)

☐ None of the above, but I think that it started, when I had_________________

☐ Unknown

**… or do you have a history of**

□ Migraine

□ Epilepsy

□ Memory problems/confusion/concentration problems

□ Mental health problems

□ Problems with gait or tremor

□ Hormonal disease

□ Autoimmune disease

□ other [-> if other] please specify ………………..

FEMALE PARTICIPANTS ONLY

□ Are your currently pregnant YES/NO

**8) – CN V question**

**8. Have you experienced sensations of burning, cooling or tingling in your nose?**

☐ Yes ☐ No

If yes, how often have you experienced this?

☐ every day

☐ XX days per week

☐ XX days per month

☐ at the same time as my smell problem

**9) TASTE DOMAIN**

**9) Is your sense of true taste (perception of something salty/sweet/sour/bitter/savoury as perceived on the tongue) altered?**

☐ Yes (-> 9a and 9b), etc.) ☐ No (-> ‘no problem’ [according to SNOT-22 scale] -> continue with 10)

**9a) How would you rate this problem?** [according to SNOT-22 scale]

1 – very mild problem

2 – mild or slight problem

3 – moderate problem

4 – severe problem

5 – problem as bad as it can be

**9b) Is your sense of true taste (perception of something salty/sweet/sour/bitter/savoury as perceived on the tongue)**

☐ reduced (weakened)

☐ distorted

☐ reduced (weakened) and distorted

**9c) When did this problem start? If you are not certain, please give your best estimate.**

☐ MM/YYYY

**9d) Please indicate which box applies to how your taste problem (problem with perception of something salty/sweet/sour/bitter/savoury as perceived on the tongue) changed since the time of onset:**

|  | Unchanged | Improved | Worsened | Fluctuates (sometimes better, then worse again) |  |
| --- | --- | --- | --- | --- | --- |
| Taste problem | ☐ | ☐ | ☐ | ☐ |  |

**9e) Please indicate which box applies to how your taste problem (problem with perception of something salty/sweet/sour/bitter/savoury as perceived on the tongue) changed over the last three months (please answer if the problem persisted for more than 3 months):**

|  | Unchanged | Improved | Worsened | Fluctuates (sometimes better, then worse again) |  |
| --- | --- | --- | --- | --- | --- |
| Taste problem | ☐ | ☐ | ☐ | ☐ |  |

**9f) For each of the following taste qualities, indicate whether your perception of it is normal, diminished, absent, distorted when you eat and drink:**

|  | Normal | Reduced (weakened) | Absent | Distorted | unsure |
| --- | --- | --- | --- | --- | --- |
| Sweet | ☐ | ☐ | ☐ | ☐ | ☐ |
| Salty | ☐ | ☐ | ☐ | ☐ | ☐ |
| Sour | ☐ | ☐ | ☐ | ☐ | ☐ |
| Bitter | ☐ | ☐ | ☐ | ☐ | ☐ |
| Savoury/umami | ☐ | ☐ | ☐ | ☐ | ☐ |

**9g)     How would you rate your perception of true taste (perception of something salty/sweet/sour/bitter/savoury as perceived on the tongue) today?**

0 (completely lost/no perception of taste), 1, 2, 3, 4, 5, 6, 7, 8, 9, 10 (completely normal perception of taste)

**9h) Do you notice a metallic taste?**

☐ Yes ☐ No

**9i) Do you feel burning in your mouth?**

☐ Yes ☐ No

**10) QUALITY OF LIFE DOMAIN AND RELATED DOMAINS**

**[final list of items to be included will be determined by results of planned Delphi cycle]**

**How would you rate how this problem has affected your quality of life?**

0 - no problem

1 – very mild problem

2 – mild or slight problem

3 – moderate problem

4 – severe problem

5 – problem as bad as it can be

If ‘no problem’ -> finish

If any other answer -> go to below

**Below you will find a list of social/emotional consequences of your smell or taste disorder. We would like to know more about these problems and would appreciate you answering the following question to the best of your ability. There are no right or wrong answers, and only you can provide us with this information. Please rate the items, as they have been over the past two weeks. Thank you for your participation.**

| **Q** | **Domain** | **Considering how severe the problem is when you experience it, please rate each item below on how ‘bad’ it is by circling the number that corresponds with how you feel using this scale** | **No problem** | **Very mild problem** | **Mild or slight problem** | **Moderate problem** | **Severe problem** | **Problem as bad as it can be** |
| --- | --- | --- | --- | --- | --- | --- | --- | --- |
| 1 | Social and Relationship domain | Feeling isolated and excluded | 0 | 1 | 2 | 3 | 4 | 5 |
| 2 |  | Having problems with taking part in exercise and strenuous activities like running, etc. | 0 | 1 | 2 | 3 | 4 | 5 |
| 3 |  | Visiting family and friends less often | 0 | 1 | 2 | 3 | 4 | 5 |
| 4 |  | Experiencing problems with intimate physical relationships, such as kissing and intercourse, and/or feeling less attracted to my partner | 0 | 1 | 2 | 3 | 4 | 5 |
| 5 | Food domain | Appetite | 0 | 1 | 2 | 3 | 4 | 5 |
| 6 |  | Visiting restaurants less often than used to | 0 | 1 | 2 | 3 | 4 | 5 |
| 7 |  | Preparing food | 0 | 1 | 2 | 3 | 4 | 5 |
| 8 |  | Not enjoying drinks or food as much as used to or avoiding certain foods. | 0 | 1 | 2 | 3 | 4 | 5 |
| 9 |  | A change of body weight | 0 | 1 | 2 | 3 | 4 | 5 |
| *-> If you have ticked ‘no problem’ -> move to next question*  *->If you have ticked anything else other than ‘no problem’ ->*  What was the reason for the change of your weight?  ☐ Because of smell loss or reduced (weakened) sense of smell  ☐ Because of smell distortion  ☐ Because of phantom smell  ☐ Because of loss of taste or reduced/weakened sense of taste  ☐ Other reason – please specify…  Please describe your change of weight further.  ☐ I have gained weight -> if yes. How much weight (in XX kg or XX lb) did you gain in the last 3 months?  ☐ I have lost weight-> if yes. How much weight (in XX kg or XX lb) did you lose in the last 3 months?   - Because of the changes in my sense of smell or taste, I avoid certain foods. (please circle appropriate answer)   Yes No  If yes-> Which foods?  □ coffee  □ mint  □ onion  □ meat  □ egg  □ chocolate  □ garlic  □ other (please list all other foods which you avoid) ……………………….. | | | | | | | | |
| 10 | Emotional domain | Emotionally affected by smell/taste problem | 0 | 1 | 2 | 3 | 4 | 5 |
| 11 |  | Feeling angry/annoyed | 0 | 1 | 2 | 3 | 4 | 5 |
| 12 |  | Feeling sad | 0 | 1 | 2 | 3 | 4 | 5 |
| 13 |  | Thoughts focusing on what’s lacking | 0 | 1 | 2 | 3 | 4 | 5 |
| 14 |  | Feeling anxious | 0 | 1 | 2 | 3 | 4 | 5 |
| 15 |  | Feeling embarrassed | 0 | 1 | 2 | 3 | 4 | 5 |
| 16 |  | Worried that unable to get used to the changes in sense of smell/taste ever | 0 | 1 | 2 | 3 | 4 | 5 |
| 17 | Hygiene domain | Problems with personal hygiene | 0 | 1 | 2 | 3 | 4 | 5 |
| 18 |  | Inability to sense own body odour/smell. | 0 | 1 | 2 | 3 | 4 | 5 |
| 19 |  | Inability to smell spilled food or dirt in house or flat. | 0 | 1 | 2 | 3 | 4 | 5 |
| 20 | Danger domain | Inability to smell smoke or gas. | 0 | 1 | 2 | 3 | 4 | 5 |
| 21 |  | Inability to smell spoiled food. | 0 | 1 | 2 | 3 | 4 | 5 |

------------------------

**11) Additional Questions (optional)**

**11) Please answer the following 5 questions as openly and honestly as possible?**

**1 = strongly disagree**

**2 = disagree**

**3 = slightly disagree**

**4 = neither agree nor disagree**

**5 = slightly agree**

**6 = agree**

**7 = strongly agree**

| *1 In most ways my life is close to my ideal.* | 1 2 3 4 5 6 7 |
| --- | --- |
| *2 The conditions of my life are excellent. (‘My life’s great!’)* | 1 2 3 4 5 6 7 |
| *3 I am satisfied with my life.* | 1 2 3 4 5 6 7 |
| *4 So far I have achieved the important things I want in life.* | 1 2 3 4 5 6 7 |
| *5 If I could live my life over, I would change almost nothing.* | 1 2 3 4 5 6 7 |

**(31–35** Extremely satisfied – *Congratulations on finding life rewarding!***26–30** Satisfied

**21–25** Slightly satisfied – *So what areas would you like to improve?*

**20** Neutral

**15–19** Slightly dissatisfied – *Psychological wealth includes some negative emotions, so not to worry*

**10–14** Dissatisfied – *Some bad events going on? What do you need to do to improve your reactions to them?*

**5–9** Extremely dissatisfied)

**Do you suffer from any of the following problems?**

☐ Difficulty thinking or concentrating (sometimes referred to as “brain fog”)

☐ Persistent headache

☐ Pins and needles sensation

☐ sleep problems

☐ dizziness when you stand up (light-headedness)

☐ depression or anxiety

**Please provide your feedback on this questionnaire (i.e. Were the questions clear to you? Did you have any difficulties filling out this form? Do you have any suggestions?):** ____________________________________________________________________________________________________________________________________________

Thank you very much for taking the time to complete this questionnaire.
